# Increased Levels of Endothelin-1 in Cerebrospinal Fluid Are a Marker of Poor Visual Recovery after Optic Neuritis in Multiple Sclerosis Patients

**DOI:** 10.1155/2019/9320791

**Published:** 2019-09-10

**Authors:** Massimiliano Castellazzi, Giuseppe Lamberti, Maria Vittoria Resi, Eleonora Baldi, Luisa Maria Caniatti, Giuditta Galante, Paolo Perri, Maura Pugliatti

**Affiliations:** ^1^Department of Biomedical and Specialty Surgical Sciences, University of Ferrara, Ferrara, Italy; ^2^Interdepartmental Research Center for the Study of Multiple Sclerosis and Inflammatory and Degenerative Diseases of the Nervous System, University of Ferrara, Ferrara, Italy; ^3^Department of Neuroscience and Rehabilitation, S. Anna University Hospital, Ferrara, Italy; ^4^Department of Specialty Surgery, S. Anna University Hospital, Ferrara, Italy; ^5^School of Medicine, University of Ferrara, Italy

## Abstract

**Background:**

Multiple sclerosis (MS), a chronic inflammatory and degenerative disease of the central nervous system, typically features immune-mediated focal demyelination and secondary axonal degeneration. Cerebral hypoperfusion of the normal-appearing white matter (NAWM) has been reported in MS patients and may be mediated by elevated levels of endothelin-1 (ET-1), a most potent vasoconstrictive peptide released from reactive astrocytes in MS focal lesions. Optic neuritis (ON) is one of the most frequent manifestations of MS and also shows peripapillary vascular hypoperfusion in combination with disc swelling.

**Aims:**

We aimed to compare serum and cerebrospinal fluid (CSF) levels of ET-1 as a potential prognostic marker of MS-ON in two groups of patients differing for severity of MS-ON clinical presentation.

**Materials and Methods:**

A cross-sectional study to compare serum and CSF levels of ET-1 between patients with clinically aggressive MS-ON (A-MS-ON) and nonaggressive MS-ON (NA-MS-ON) according to conventional ophthalmological criteria, including optical coherence tomography. CSF and serum concentrations of ET-1 were measured using a commercially available ELISA method.

**Results:**

Sixteen patients consecutively referred to the Units of Neurology for visual disturbances attributable to MS were recruited, 11 (69%) patients with A-MS-ON and 5 (31%) with NA-MS-ON. Median CSF ET-1 levels and CSF/serum ET-1 quotient were significantly higher in patients with A-MS-ON (0.30 vs. 0.56 ng/ml) as compared to NA-MS-ON (0.16 vs. 0.16).

**Conclusions:**

Severity and failure in the recovery from ON in MS patients may depend from vascular hypoperfusion of the optic nerve induced by high intrathecally produced ET-1, a potential prognostic marker of ON recovery in MS. The detection of CSF ET-1 levels may allow identifying groups of ON patients potentially benefitting from treatment with ET-1 antagonists (e.g., bosentan).

## 1. Introduction

Multiple sclerosis (MS) is a chronic inflammatory and degenerative disease of the central nervous system (CNS) and the most frequent cause of neurological disability in young adulthood. Immune-mediated focal demyelination and secondary axonal degeneration with T-cell-mediated inflammatory response against CNS myelin are the disease pathological hallmark.

More recently, cerebral hypoperfusion of the normal-appearing white matter (NAWM) at perfusion-weighted magnetic resonance imaging (MRI) studies has been reported in patients with both relapsing-remitting and progressive MS at an early stage of the disease [1, 2] and in association with cognitive decline and fatigue [3]. By promoting mitochondrial dysfunction, oxidative stress, and axonal loss [4], blood perfusion of the gray matter is found significantly decreased in MS patients as compared with control subjects, and hypoperfusion is directly associated with degeneration and atrophy [5].

Cerebral hypoperfusion in MS has been hypothesized to be mediated by elevated levels of endothelin-1 (ET-1), a potent vasoconstrictive peptide released in the cerebral circulation from reactive astrocytes in MS focal lesions [6] and actively implicated in modulating activities of neurons and glia [7, 8].

ET-1-related reactive astrogliosis has been reported for neurodegeneration in Alzheimer's disease [9], traumatic brain injury [10], and stroke [11]. Optic neuritis (ON) is one of the most frequent manifestations of MS (Optic Neuritis Study Group 2008). Among the typical MS-ON manifestations are subacute monocular visual loss associated with pain during eye movement, visual loss or blurring, and dyschromatopsia. Acute MS-ON also shows peripapillary vascular hypoperfusion in combination with disc swelling [12]. Persistent demyelination [13] and axonal loss account for incomplete or poor visual recovery in MS-ON.

We assessed the levels of ET-1 in the cerebrospinal fluid (CSF) and in serum with a special focus on ON which occurred in MS patients as a potential prognostic marker of visual outcome.

## 2. Materials and Methods

We designed a cross-sectional study to compare serum and CSF levels of ET-1 between patients with clinically aggressive MS-ON (A-MS-ON) and patients with nonaggressive MS-ON (NA-MS-ON). For the study purposes, subjects with a diagnosis of ON were regarded as “aggressive”(A-MS-ON) if at least one of the following criteria was met: (i) visual acuity of 5/10 or less, (ii) visual acuity greater than 5/10 but possible through residual receptive fields, (iii) paracentral scotoma with 1 point less than 10 dB or at least 3 contiguous points less than 4 dB according to Heijl-Krakau criteria, (iv) a deep and wide defect of the papillomacular bundle at optical coherence tomography (OCT) (structural criterion), (v) concomitant inflammatory and ischemic changes of postchiasmatic visual pathways, and (iv) impairment of oculomotor pathways with diplopia concomitant with visual deficit onset. MS-ON presentation which did not meet any of the above criteria was defined “nonaggressive” (NA-MS-ON).

A history of comorbidity with special regard to cardiovascular disorders and metabolic syndromes was collected from the study participants.

The study was approved by the local Committee for Medical Ethics in Research (Protocol no. 151197, 21 January 2016), and written informed consent was obtained from all participants.

### 2.1. Sample Handling

Paired CSF and serum samples were collected as a part of the diagnostic workup and measured under the same conditions. Cell-free CSF and serum were (i) obtained after centrifugation at 2,000 *g* at 20°C for 15 minutes, (ii) collected under sterile conditions in aliquots of 500 *μ*l, (iii) coded, and (iv) stored at -80°C until assay.

### 2.2. Optical Coherence Tomography

Optical coherence tomography (OCT) scans were performed without pupillary dilation and according to standardized operating procedures, on Stratus TD-OCT (M. 3000, software version 4.0; Carl Zeiss Meditec, Dublin, CA). Peripapillary retinal nerve fiber layer (pRNFL) thickness was measured according to the fast pRNFL thickness protocol, i.e., three consecutive 3.4 lm diameter circular scans (256 A-scans/B-scan) which were centered on the optic disc.

An image quality signal strength of less than 7 (on a scale of 1–10) in optic disc and macular scans and the presence of artifacts were exclusion criteria.

### 2.3. Cerebrospinal Fluid and Serum Levels of Endothelin-1

CSF and serum concentrations of ET-1 were measured using a commercially available ELISA method (R&D Systems, Minneapolis, MN; Quantikine ELISA, cat. num. DET100). CSF samples were analyzed undiluted, while serum samples were diluted 1 : 2. As indicated by the manufacturer, the range of determinability ranged from 0.4 to 25 pg/ml. The assay had an intra-assay precision with a percent coefficient of variation (CV) of less than 5% and an interassay accuracy of less than 10%. The CSF/serum ET-1 quotient was calculated for all patients.

### 2.4. Statistical Analysis

Frequencies (percent) were reported for categorical variables and median and interquartile range (IQR) for continuous variables. Differences were searched with the chi-square test for categorical variables and the Mann-Whitney nonparametric comparison for continuous variables. Significance was set at *p* value < 0.05, on a two-tailed test. The Statistical Package for the Social Sciences (SPSS) version 19 for Windows and OSX (SPSS Inc., IBM, Somers, New York, USA) was used for statistical analysis.

## 3. Results

The study was conducted on 16 patients consecutively referred to the Units of Neurology for visual disturbances attributable to ON in MS. Eleven (69%) patients received a diagnosis of aggressive ON (A-MS-ON) and 5 (31%) of nonaggressive ON (NA-MS-ON) according to given definition (see Materials and Methods). The main clinical and demographic features of the study population are reported in Table 1. Sex distribution differed from the two groups with no male population represented in the NA-MS-ON group.

Median CSF ET-1 levels and CSF/serum ET-1 quotient were significantly higher in patients with A-MS-ON (0.30 vs. 0.56 ng/ml) as compared to NA-MS-ON (0.16 vs. 0.16) (Table 2).

No differences were found for other CSF parameters, such as albumin quotient, CSF total proteins, CSF cell count, and presence of IgG oligoclonal bands (data not shown).

None of the study participants presented with comorbidities.

## 4. Discussion

Optic neuritis (ON) is the clinical onset manifestation of MS for 25% of patients, and about 70% of MS patients present ON during the disease course [14]. Recovery from typical MS-ON occurs within few days or weeks from symptom onset and is complete in the majority of patients. Incomplete recovery with visual acuity of 20/40 or poorer occurs in about 10% of patients [15, 16] likely attributable to persistent demyelination [13] and axonal loss.

We aimed to detect and compare CSF with serum levels of ET-1 as a potential marker of visual outcome in two groups of patients with MS-ON differing for severity which was clinically and instrumentally assessed. For the first time to our knowledge, we show that aggressive ON in MS features significantly higher CSF levels of ET-1 as compared to nonaggressive ON and that such high ET-1 levels depend on intrathecal synthesis, as demonstrated with the increased ET-1 quotient.

Elevated ET-1 levels in the plasma and hypoperfusion of the ophthalmic artery and its retrobulbar branches were found in MS patients as compared to a group of controls [17], but a specific correlation with a history of ON was not investigated. Also, ET-1 blood levels were found increased in patients with ON, but a relationship with visual performance was not assessed [18].

Reactive astrocytes in MS demyelinating plaques are the potential source of ET-1, most likely in response to proinflammatory stimuli (e.g., TNF-*α* and IL-1*β*) during acute inflammation [19]. Acting predominantly on the ET_A_ receptors expressed in the CNS, ET-1 induces vasoconstriction. This, however, is partly counterbalanced by the action of ET-1 on the ET_B_ receptors with consequent synthesis of nitric oxide with vasodilating action which in turn inhibits ET-1 synthesis. In a subpopulation of MS patients, the balance between vasoconstriction and vasodilation was found to be in favour of the former, yielding hypoxic-ischemic processes determining axonal demyelination and retinal ganglion cell apoptosis [12].

In the ocular system, ET-1 generates vasoconstriction only in the presence of an altered blood-brain or blood-retinal barrier through direct access to smooth muscle [20]. Because the head of the optic nerve has no proper blood-brain barrier, this structure shows even greater vulnerability to ET-1-induced hypoperfusion and ischemic change [21].

In our study, no difference was detected between the times of ON presentation in relation to MS diagnosis in the aggressive vs. nonaggressive ON: ON was the first clinical symptom of MS in 56.2% of A-MS-ON and 50% of NA-MS-ON, with the remaining ONs presenting during the disease course. ET-1 levels in serum, CSF, and the ET-1 quotient did not differ by timing of ON presentation within MS (data not shown).

We looked at a possible correlation between the severity of ON presentation and clinical response to corticosteroid iv treatment in an ON acute phase. Previous studies have also shown that dexamethasone, a synthetic corticosteroid, on the one hand induces an increase of ET-1 release by nonpigmented ciliary epithelial cells, and on the other hand, it suppresses the expression of the ET-B receptor with a consequent decrease in nitric oxide synthesis induced by ET-1 [22]. This combined effect of corticosteroids and increased levels of ET-1 favours vasoconstriction and consequent hypoxic-ischemic tissue damage in these subgroups. Our small study population however did not allow us to discern between responders and nonresponders to an iv corticosteroid treatment which appeared to be efficacious in most cases, irrespective of ON severity and ET-1 levels.

The main study limitations are the small sample size and its retrospective nature. An estimation of incidence or prevalence of higher CSF ET-1 levels in ONs is needed for sample size calculation, but to our knowledge, this is not available from literature.

Furthermore, potential confounding in these findings may have been introduced with the use of OCT retinal measures reflecting hypoperfusion, such as a lower pRNFL or a lower ganglion cell and inner plexiform layer (GCIP) thickness, which have been reported to predict *per se* significant worsening of MS 10-year disability [23]. In fact, whether poor visual recovery from MS-ON depends on retinal layer at OCT or whether a concomitant history of ON contributes to disability progression is still debated [24, 25].

## 5. Conclusions

Poor visual recovery from ON in MS patients may depend on blood hypoperfusion of the optic nerve induced by high intrathecally produced ET-1, a potent vasoconstrictor, and thus a potential prognostic marker of ON outcome in MS.

The detection of CSF ET-1 levels may allow to identify groups of ON patients potentially benefitting from treatment with ET-1 antagonists (e.g., bosentan) [6, 26] rather than from high doses of iv corticosteroids commonly used to treat acute visual exacerbation in MS. Further prospective research, including investigating other reactive astrocyte-related biomarkers (e.g., IL-6 and prostaglandins), is needed to corroborate these evidences.

## Figures and Tables

**Table 1:** Clinical and demographic features of the study population.

	A-MS-ON *N* = 11	NA-MS-ON *N* = 5	*p*
Sex (F); *N* (%)	7 (63.6)	5 (100)	*0.245*
Mean (SD) age at study time (years)	36.7 (10.5)	40.2 (5.9)	*0.495*
Age range at study time (years)	20-53	35-48	
Mean (SD) age of MS-ON onset (years)	35.5 (9.8)	40.0 (5.6)	*0.335*
Age range at MS-ON onset (years)	20-52	35-47	
ON at MS onset; *N* (%)	7 (63.6)	3 (60.0)	*1.000*

Fisher's exact test and the Mann-Whitney *u*-test were used to compare categorical and continous variables, respectively. A-MS-ON: aggressive optic neuritis in multiple sclerosis; NA-MS-ON: nonaggressive optic neuritis in multiple sclerosis (see text for definition); SD: standard deviation.

**Table 2:** Mean (SD) concentrations of serum and CSF ET-1 (pg/ml) in patients with aggressive vs. nonaggressive MS-ON phenotype.

	A-MS-ON *N* = 11	NA-MS-ON *N* = 5	*p*
Median (IQR) serum ET-1: pg/ml	0.65 (0.49-0.81)	0.82 (0.59-1.17)	*0.3059^a^*
Median (IQR) CSF ET-1: pg/ml	0.30 (0.22-0.55)	0.16 (0.05-0.23)	*0.008^a^*
Median (IQR) CSF/serum ET-1 quotient	0.56 (0.34-0.68)	0.16 (0.06-0.33)	*0.0034^a^*

^a^Mann-Whitney test for nonparametric data; A-MS-ON: aggressive optic neuritis in multiple sclerosis (see text for definition); CSF: cerebrospinal fluid; ET-1: endothelin-1; NA-MS-ON: nonaggressive optic neuritis in multiple sclerosis (see text for definition).

## Data Availability

Data are available upon reasonable request.

## References

[1] Adhya S., Johnson G., Herbert J. (2006). Pattern of hemodynamic impairment in multiple sclerosis: dynamic susceptibility contrast perfusion MR imaging at 3.0 T. *NeuroImage*.

[2] Law M., Saindane A. M., Ge Y. (2004). Microvascular abnormality in relapsing-remitting multiple sclerosis: perfusion MR imaging findings in normal-appearing white matter. *Radiology*.

[3] D'haeseleer M., Hostenbach S., Peeters I. (2015). Cerebral hypoperfusion: a new pathophysiologic concept in multiple sclerosis?. *Journal of Cerebral Blood Flow and Metabolism*.

[4] Aliev G., Smith M. A., Obrenovich M. E., de la Torre J. C., Perry G. (2003). Role of vascular hypoperfusion-induced oxidative stress and mitochondria failure in the pathogenesis of Alzheimer disease. *Neurotoxicity Research*.

[5] Marshall O., Lu H., Brisset J. C. (2014). Impaired cerebrovascular reactivity in multiple sclerosis. *JAMA Neurology*.

[6] D'Haeseleer M., Beelen R., Fierens Y. (2013). Cerebral hypoperfusion in multiple sclerosis is reversible and mediated by endothelin-1. *Proceedings of the National Academy of Sciences of the United States of America*.

[7] Blomstrand F., Giaume C., Hansson E., Ronnback L. (1999). Distinct pharmacological properties of ET-1 and ET-3 on astroglial gap junctions and Ca^2+^ signaling. *American Journal of Physiology*.

[8] Stanimirovic D. B., Ball R., Mealing G., Morley P., Durkin J. P. (1995). The role of intracellular calcium and protein kinase C in endothelin-stimulated proliferation of rat type I astrocytes. *Glia*.

[9] Zhang W. W., Badonic T., Hoog A. (1994). Astrocytes in Alzheimer’s disease express immunoreactivity to the vaso-constrictor endothelin-1. *Journal of the Neurological Sciences*.

[10] Rogers S. D., Peters C. M., Pomonis J. D., Hagiwara H., Ghilardi J. R., Mantyh P. W. (2003). Endothelin B receptors are expressed by astrocytes and regulate astrocyte hypertrophy in the normal and injured CNS. *Glia*.

[11] Barone F. C., Globus M. Y. T., Price W. J. (1994). Endothelin levels increase in rat focal and global ischemia. *Journal of Cerebral Blood Flow & Metabolism*.

[12] Chen T. C., Yeh C. Y., Lin C. W. (2017). Vascular hypoperfusion in acute optic neuritis is a potentially new neurovascular model for demyelinating diseases. *PLoS One*.

[13] Raz N., Dotan S., Chokron S., Ben-Hur T., Levin N. (2012). Demyelination affects temporal aspects of perception: an optic neuritis study. *Annals of Neurology*.

[14] The Optic Neuritis Study Group (2008). Multiple sclerosis risk after optic neuritis: final optic neuritis treatment trial follow-up. *Archives of Neurology*.

[15] Beck R. W., Cleary P. A. (1993). Optic neuritis treatment trial. One-year follow-up results. *Archives of Ophthalmology*.

[16] Beck R. W., Cleary P. A., Backlund J. Y. C. (1994). The course of visual recovery after optic neuritis. Experience of the Optic Neuritis Treatment Trial. *Ophthalmology*.

[17] Pache M., Kaiser H. J., Akhalbedashvili N. (2003). Extraocular blood flow and endothelin-1 plasma levels in patients with multiple sclerosis. *European Neurology*.

[18] Haufschild T., Shaw S. G., Kaiser H. J., Flammer J. (2003). Transient raise of endothelin-1 plasma level and reduction of ocular blood flow in a patient with optic neuritis. *Ophthalmologica*.

[19] Konsman J. P., Drukarch B., Van Dam A. M. (2007). (Peri)vascular production and action of pro-inflammatory cytokines in brain pathology. *Clinical Science*.

[20] Strenn K., Matulla B., Wolzt M. (1998). Reversal of endothelin-1-induced ocular hemodynamic effects by low-dose nifedipine in humans. *Clinical Pharmacology and Therapeutics*.

[21] Hofman P., Hoyng P., vanderWerf F., Vrensen G. F. J. M., Schlingemann R. O. (2001). Lack of blood-brain barrier properties in microvessels of the prelaminar optic nerve head. *Investigative Ophthalmology & Visual Science*.

[22] Zhang X., Clark A. F., Yorio T. (2003). Interactions of endothelin-1 with dexamethasone in primary cultured human trabecular meshwork cells. *Investigative Ophthalmology & Visual Science*.

[23] Rothman A., Murphy O. C., Fitzgerald K. C. (2019). Retinal measurements predict 10-year disability in multiple sclerosis. *Annals of Clinical Translational Neurology*.

[24] Behbehani R., Abu Al-Hassan A., Al-Salahat A., Sriraman D., Oakley J. D., Alroughani R. (2017). Optical coherence tomography segmentation analysis in relapsing remitting versus progressive multiple sclerosis. *PLoS One*.

[25] Petzold A., Balcer L. J., Calabresi P. A. (2017). Retinal layer segmentation in multiple sclerosis: a systematic review and meta-analysis. *The Lancet Neurology*.

[26] Hostenbach S., Pauwels A., Michiels V. (2019). Role of cerebral hypoperfusion in multiple sclerosis (ROCHIMS): study protocol for a proof-of-concept randomized controlled trial with bosentan. *Trials*.

